# Supplementary material for: Modeling glioblastoma heterogeneity as a dynamic network of cell states
Source: Mol Syst Biol. 2021 Sep 16;17(9):e10105. doi: 10.15252/msb.202010105 (PMC8444284; doi:10.15252/msb.202010105)
Supplement: Supplementary file 6 — Source Data for Figure 5 [file MSB-17-e10105-s004.zip › Figure5A_sourcedata/GSEA_3071/nosigdb_stateV.GseaPreranked.1622795159954/RICHARDS_NOWAKOWSKI_SCIENCE_2017_MGE_IPC2_UPREG.html]

Details for gene set RICHARDS\_NOWAKOWSKI\_SCIENCE\_2017\_MGE\_IPC2\_UPREG[GSEA]

|  || Dataset | state43071 |
| Phenotype | NoPhenotypeAvailable |
| Upregulated in class | na\_pos |
| GeneSet | RICHARDS\_NOWAKOWSKI\_SCIENCE\_2017\_MGE\_IPC2\_UPREG |
| Enrichment Score (ES) | 0.6823572 |
| Normalized Enrichment Score (NES) | 4.635448 |
| Nominal p-value | 0.0 |
| FDR q-value | 0.0 |
| FWER p-Value | 0.0 |
Table: GSEA Results Summary

  

Fig 1: Enrichment plot: RICHARDS\_NOWAKOWSKI\_SCIENCE\_2017\_MGE\_IPC2\_UPREG      
 Profile of the Running ES Score & Positions of GeneSet Members on the Rank Ordered List

  

| PROBE | GENE SYMBOL | GENE\_TITLE | RANK IN GENE LIST | RANK METRIC SCORE | RUNNING ES | CORE ENRICHMENT || 1 | HIST1H1B |  |  | 0 | 2.500 | 0.0064 | Yes |
| 2 | HIST1H4C |  |  | 1 | 2.442 | 0.0126 | Yes |
| 3 | ATAD2 |  |  | 2 | 2.214 | 0.0182 | Yes |
| 4 | ESCO2 |  |  | 3 | 2.193 | 0.0238 | Yes |
| 5 | FAM111B |  |  | 4 | 2.012 | 0.0289 | Yes |
| 6 | HMGB2 |  |  | 5 | 2.011 | 0.0340 | Yes |
| 7 | NUSAP1 |  |  | 6 | 2.001 | 0.0391 | Yes |
| 8 | HIST1H1C |  |  | 7 | 1.976 | 0.0441 | Yes |
| 9 | PCNA |  |  | 10 | 1.908 | 0.0485 | Yes |
| 10 | TOP2A |  |  | 14 | 1.842 | 0.0526 | Yes |
| 11 | HIST1H1E |  |  | 15 | 1.830 | 0.0573 | Yes |
| 12 | MCM4 |  |  | 17 | 1.815 | 0.0617 | Yes |
| 13 | DHFR |  |  | 18 | 1.795 | 0.0662 | Yes |
| 14 | HIST1H1D |  |  | 19 | 1.786 | 0.0708 | Yes |
| 15 | C21orf58 |  |  | 20 | 1.772 | 0.0753 | Yes |
| 16 | NCAPG |  |  | 21 | 1.760 | 0.0797 | Yes |
| 17 | RAD51AP1 |  |  | 22 | 1.757 | 0.0842 | Yes |
| 18 | FAM111A |  |  | 24 | 1.738 | 0.0884 | Yes |
| 19 | SYNE2 |  |  | 25 | 1.732 | 0.0928 | Yes |
| 20 | CENPF |  |  | 28 | 1.705 | 0.0967 | Yes |
| 21 | TYMS |  |  | 30 | 1.683 | 0.1008 | Yes |
| 22 | CCNE2 |  |  | 32 | 1.664 | 0.1048 | Yes |
| 23 | RRM2 |  |  | 34 | 1.660 | 0.1088 | Yes |
| 24 | MYBL2 |  |  | 35 | 1.651 | 0.1130 | Yes |
| 25 | CENPU |  |  | 36 | 1.645 | 0.1172 | Yes |
| 26 | CDCA7L |  |  | 39 | 1.619 | 0.1209 | Yes |
| 27 | ASCL1 |  |  | 43 | 1.604 | 0.1244 | Yes |
| 28 | MKI67 |  |  | 45 | 1.598 | 0.1282 | Yes |
| 29 | BRCA1 |  |  | 46 | 1.592 | 0.1323 | Yes |
| 30 | HELLS |  |  | 48 | 1.588 | 0.1361 | Yes |
| 31 | MCM3 |  |  | 49 | 1.586 | 0.1401 | Yes |
| 32 | XRCC2 |  |  | 50 | 1.574 | 0.1441 | Yes |
| 33 | KIF15 |  |  | 52 | 1.568 | 0.1479 | Yes |
| 34 | FOXM1 |  |  | 53 | 1.550 | 0.1519 | Yes |
| 35 | PBK |  |  | 54 | 1.536 | 0.1558 | Yes |
| 36 | EZH2 |  |  | 55 | 1.534 | 0.1597 | Yes |
| 37 | GINS2 |  |  | 57 | 1.528 | 0.1633 | Yes |
| 38 | DTL |  |  | 60 | 1.503 | 0.1667 | Yes |
| 39 | GTSE1 |  |  | 66 | 1.491 | 0.1695 | Yes |
| 40 | E2F8 |  |  | 67 | 1.488 | 0.1733 | Yes |
| 41 | CDK1 |  |  | 69 | 1.488 | 0.1769 | Yes |
| 42 | INSM1 |  |  | 72 | 1.467 | 0.1802 | Yes |
| 43 | SMC2 |  |  | 73 | 1.466 | 0.1839 | Yes |
| 44 | SPC25 |  |  | 76 | 1.442 | 0.1872 | Yes |
| 45 | CHAF1A |  |  | 77 | 1.441 | 0.1908 | Yes |
| 46 | UBE2T |  |  | 79 | 1.432 | 0.1943 | Yes |
| 47 | FANCD2 |  |  | 81 | 1.423 | 0.1977 | Yes |
| 48 | FBXO5 |  |  | 82 | 1.422 | 0.2013 | Yes |
| 49 | CLSPN |  |  | 84 | 1.417 | 0.2047 | Yes |
| 50 | MCM7 |  |  | 88 | 1.400 | 0.2076 | Yes |
| 51 | MCM8 |  |  | 89 | 1.397 | 0.2112 | Yes |
| 52 | MCM10 |  |  | 92 | 1.389 | 0.2143 | Yes |
| 53 | DEK |  |  | 94 | 1.384 | 0.2176 | Yes |
| 54 | FBLN1 |  |  | 95 | 1.383 | 0.2211 | Yes |
| 55 | CDCA2 |  |  | 96 | 1.382 | 0.2246 | Yes |
| 56 | RRM1 |  |  | 99 | 1.364 | 0.2277 | Yes |
| 57 | DLX2 |  |  | 104 | 1.357 | 0.2303 | Yes |
| 58 | TOPBP1 |  |  | 108 | 1.350 | 0.2331 | Yes |
| 59 | POLD3 |  |  | 111 | 1.340 | 0.2361 | Yes |
| 60 | KIFC1 |  |  | 113 | 1.338 | 0.2393 | Yes |
| 61 | CENPM |  |  | 114 | 1.337 | 0.2427 | Yes |
| 62 | NCAM1 |  |  | 117 | 1.334 | 0.2457 | Yes |
| 63 | ZNF704 |  |  | 120 | 1.329 | 0.2486 | Yes |
| 64 | LMO1 |  |  | 122 | 1.326 | 0.2518 | Yes |
| 65 | CDCA5 |  |  | 123 | 1.326 | 0.2552 | Yes |
| 66 | SMC4 |  |  | 127 | 1.320 | 0.2579 | Yes |
| 67 | DLX1 |  |  | 128 | 1.314 | 0.2612 | Yes |
| 68 | BARD1 |  |  | 132 | 1.310 | 0.2640 | Yes |
| 69 | MMS22L |  |  | 134 | 1.306 | 0.2671 | Yes |
| 70 | TMSB15A |  |  | 135 | 1.306 | 0.2704 | Yes |
| 71 | FEN1 |  |  | 139 | 1.296 | 0.2731 | Yes |
| 72 | DLX5 |  |  | 140 | 1.294 | 0.2764 | Yes |
| 73 | RTKN2 |  |  | 143 | 1.285 | 0.2792 | Yes |
| 74 | LIG1 |  |  | 145 | 1.284 | 0.2823 | Yes |
| 75 | GMNN |  |  | 146 | 1.280 | 0.2855 | Yes |
| 76 | SOX2 |  |  | 147 | 1.279 | 0.2888 | Yes |
| 77 | NCAPD3 |  |  | 155 | 1.261 | 0.2905 | Yes |
| 78 | NCAPD2 |  |  | 157 | 1.252 | 0.2935 | Yes |
| 79 | CCNF |  |  | 158 | 1.250 | 0.2967 | Yes |
| 80 | MIS18BP1 |  |  | 159 | 1.247 | 0.2998 | Yes |
| 81 | POLQ |  |  | 160 | 1.245 | 0.3030 | Yes |
| 82 | TMPO |  |  | 161 | 1.243 | 0.3062 | Yes |
| 83 | CENPK |  |  | 162 | 1.241 | 0.3093 | Yes |
| 84 | CDKN3 |  |  | 163 | 1.239 | 0.3125 | Yes |
| 85 | LMNB1 |  |  | 165 | 1.238 | 0.3154 | Yes |
| 86 | DNMT1 |  |  | 166 | 1.237 | 0.3186 | Yes |
| 87 | KIF23 |  |  | 167 | 1.235 | 0.3217 | Yes |
| 88 | NCAPH |  |  | 168 | 1.235 | 0.3248 | Yes |
| 89 | CDCA3 |  |  | 169 | 1.230 | 0.3280 | Yes |
| 90 | RFC4 |  |  | 171 | 1.227 | 0.3309 | Yes |
| 91 | RNF168 |  |  | 175 | 1.225 | 0.3334 | Yes |
| 92 | NASP |  |  | 180 | 1.212 | 0.3356 | Yes |
| 93 | LSAMP |  |  | 186 | 1.203 | 0.3376 | Yes |
| 94 | DBF4 |  |  | 189 | 1.197 | 0.3403 | Yes |
| 95 | KIF4A |  |  | 191 | 1.193 | 0.3431 | Yes |
| 96 | NUF2 |  |  | 192 | 1.189 | 0.3461 | Yes |
| 97 | FANCI |  |  | 196 | 1.179 | 0.3485 | Yes |
| 98 | ZNF43 |  |  | 201 | 1.169 | 0.3506 | Yes |
| 99 | USP1 |  |  | 202 | 1.168 | 0.3536 | Yes |
| 100 | ZWINT |  |  | 204 | 1.166 | 0.3564 | Yes |
| 101 | RNASEH2B |  |  | 205 | 1.166 | 0.3593 | Yes |
| 102 | ASPM |  |  | 207 | 1.163 | 0.3621 | Yes |
| 103 | RBBP8 |  |  | 210 | 1.162 | 0.3646 | Yes |
| 104 | PRC1 |  |  | 211 | 1.161 | 0.3676 | Yes |
| 105 | ARL6IP1 |  |  | 212 | 1.158 | 0.3705 | Yes |
| 106 | KIF22 |  |  | 213 | 1.157 | 0.3735 | Yes |
| 107 | UBE2C |  |  | 214 | 1.154 | 0.3764 | Yes |
| 108 | SPECC1 |  |  | 216 | 1.153 | 0.3791 | Yes |
| 109 | CEP97 |  |  | 221 | 1.146 | 0.3812 | Yes |
| 110 | NCAPG2 |  |  | 225 | 1.141 | 0.3835 | Yes |
| 111 | BTG3 |  |  | 226 | 1.141 | 0.3864 | Yes |
| 112 | CDK2 |  |  | 233 | 1.134 | 0.3880 | Yes |
| 113 | RFC3 |  |  | 239 | 1.130 | 0.3899 | Yes |
| 114 | NDC80 |  |  | 240 | 1.129 | 0.3927 | Yes |
| 115 | MCM5 |  |  | 243 | 1.127 | 0.3952 | Yes |
| 116 | TPX2 |  |  | 248 | 1.122 | 0.3972 | Yes |
| 117 | BRIP1 |  |  | 249 | 1.121 | 0.4001 | Yes |
| 118 | SMC1A |  |  | 251 | 1.120 | 0.4027 | Yes |
| 119 | SP9 |  |  | 254 | 1.116 | 0.4051 | Yes |
| 120 | CDH2 |  |  | 255 | 1.115 | 0.4080 | Yes |
| 121 | RAD21 |  |  | 256 | 1.109 | 0.4108 | Yes |
| 122 | TIMELESS |  |  | 259 | 1.105 | 0.4132 | Yes |
| 123 | LINC01224 |  |  | 263 | 1.098 | 0.4153 | Yes |
| 124 | KIF14 |  |  | 272 | 1.090 | 0.4165 | Yes |
| 125 | HAUS8 |  |  | 273 | 1.089 | 0.4192 | Yes |
| 126 | BRCA2 |  |  | 274 | 1.089 | 0.4220 | Yes |
| 127 | CHD7 |  |  | 275 | 1.088 | 0.4248 | Yes |
| 128 | MAGI1 |  |  | 276 | 1.086 | 0.4275 | Yes |
| 129 | BUB1 |  |  | 277 | 1.084 | 0.4303 | Yes |
| 130 | ZBTB20 |  |  | 281 | 1.080 | 0.4324 | Yes |
| 131 | MCM6 |  |  | 282 | 1.080 | 0.4351 | Yes |
| 132 | CKAP2L |  |  | 285 | 1.078 | 0.4375 | Yes |
| 133 | HIRIP3 |  |  | 289 | 1.077 | 0.4396 | Yes |
| 134 | HIST1H3D |  |  | 290 | 1.077 | 0.4423 | Yes |
| 135 | CDK5RAP2 |  |  | 292 | 1.076 | 0.4449 | Yes |
| 136 | PRIM1 |  |  | 293 | 1.074 | 0.4476 | Yes |
| 137 | ECT2 |  |  | 295 | 1.071 | 0.4501 | Yes |
| 138 | AURKB |  |  | 297 | 1.068 | 0.4526 | Yes |
| 139 | ARHGAP11A |  |  | 303 | 1.064 | 0.4543 | Yes |
| 140 | STMN1 |  |  | 307 | 1.062 | 0.4564 | Yes |
| 141 | CCNA2 |  |  | 309 | 1.057 | 0.4588 | Yes |
| 142 | H1FX |  |  | 310 | 1.055 | 0.4615 | Yes |
| 143 | CKAP2 |  |  | 312 | 1.053 | 0.4640 | Yes |
| 144 | PSIP1 |  |  | 313 | 1.053 | 0.4667 | Yes |
| 145 | DUT |  |  | 314 | 1.050 | 0.4693 | Yes |
| 146 | PRR11 |  |  | 317 | 1.048 | 0.4716 | Yes |
| 147 | WEE1 |  |  | 322 | 1.044 | 0.4734 | Yes |
| 148 | CDCA7 |  |  | 330 | 1.038 | 0.4746 | Yes |
| 149 | TMEM106C |  |  | 334 | 1.034 | 0.4766 | Yes |
| 150 | RFC2 |  |  | 337 | 1.025 | 0.4788 | Yes |
| 151 | ZGRF1 |  |  | 340 | 1.024 | 0.4810 | Yes |
| 152 | KNTC1 |  |  | 342 | 1.023 | 0.4834 | Yes |
| 153 | WDR76 |  |  | 350 | 1.015 | 0.4845 | Yes |
| 154 | EXO1 |  |  | 354 | 1.010 | 0.4865 | Yes |
| 155 | PROM1 |  |  | 356 | 1.008 | 0.4888 | Yes |
| 156 | RBL1 |  |  | 358 | 0.999 | 0.4912 | Yes |
| 157 | KIF11 |  |  | 369 | 0.982 | 0.4916 | Yes |
| 158 | RAB3IP |  |  | 371 | 0.980 | 0.4939 | Yes |
| 159 | APOLD1 |  |  | 372 | 0.980 | 0.4964 | Yes |
| 160 | MYBL1 |  |  | 375 | 0.977 | 0.4984 | Yes |
| 161 | ILDR2 |  |  | 376 | 0.977 | 0.5009 | Yes |
| 162 | DSN1 |  |  | 377 | 0.975 | 0.5034 | Yes |
| 163 | CASP8AP2 |  |  | 379 | 0.970 | 0.5057 | Yes |
| 164 | TTK |  |  | 383 | 0.966 | 0.5075 | Yes |
| 165 | PCM1 |  |  | 386 | 0.964 | 0.5095 | Yes |
| 166 | DEPDC1B |  |  | 387 | 0.963 | 0.5120 | Yes |
| 167 | UHRF1 |  |  | 390 | 0.958 | 0.5140 | Yes |
| 168 | CIT |  |  | 391 | 0.958 | 0.5164 | Yes |
| 169 | ZNF367 |  |  | 394 | 0.956 | 0.5185 | Yes |
| 170 | YEATS4 |  |  | 397 | 0.951 | 0.5205 | Yes |
| 171 | HJURP |  |  | 406 | 0.945 | 0.5212 | Yes |
| 172 | MASTL |  |  | 407 | 0.945 | 0.5236 | Yes |
| 173 | RMI1 |  |  | 411 | 0.939 | 0.5254 | Yes |
| 174 | NEDD4L |  |  | 413 | 0.939 | 0.5276 | Yes |
| 175 | SPC24 |  |  | 414 | 0.938 | 0.5300 | Yes |
| 176 | NUDT1 |  |  | 417 | 0.936 | 0.5319 | Yes |
| 177 | DLGAP5 |  |  | 418 | 0.936 | 0.5343 | Yes |
| 178 | MAD2L1 |  |  | 421 | 0.932 | 0.5363 | Yes |
| 179 | UBR7 |  |  | 423 | 0.932 | 0.5384 | Yes |
| 180 | SAP30 |  |  | 424 | 0.930 | 0.5408 | Yes |
| 181 | CEP192 |  |  | 429 | 0.924 | 0.5423 | Yes |
| 182 | KPNA2 |  |  | 435 | 0.919 | 0.5436 | Yes |
| 183 | CENPE |  |  | 437 | 0.918 | 0.5457 | Yes |
| 184 | LBR |  |  | 438 | 0.916 | 0.5481 | Yes |
| 185 | RACGAP1 |  |  | 443 | 0.912 | 0.5496 | Yes |
| 186 | VRK1 |  |  | 445 | 0.911 | 0.5517 | Yes |
| 187 | ORC6 |  |  | 449 | 0.908 | 0.5533 | Yes |
| 188 | STIL |  |  | 455 | 0.903 | 0.5546 | Yes |
| 189 | ZNF738 |  |  | 458 | 0.901 | 0.5565 | Yes |
| 190 | POLA1 |  |  | 463 | 0.900 | 0.5580 | Yes |
| 191 | HMGN2 |  |  | 466 | 0.900 | 0.5598 | Yes |
| 192 | LSM3 |  |  | 468 | 0.892 | 0.5619 | Yes |
| 193 | MGME1 |  |  | 469 | 0.892 | 0.5642 | Yes |
| 194 | DNA2 |  |  | 470 | 0.890 | 0.5664 | Yes |
| 195 | CENPJ |  |  | 479 | 0.885 | 0.5670 | Yes |
| 196 | MND1 |  |  | 480 | 0.885 | 0.5693 | Yes |
| 197 | SPAG5 |  |  | 481 | 0.884 | 0.5715 | Yes |
| 198 | BLM |  |  | 486 | 0.879 | 0.5729 | Yes |
| 199 | MPHOSPH9 |  |  | 489 | 0.877 | 0.5747 | Yes |
| 200 | RGS12 |  |  | 499 | 0.867 | 0.5751 | Yes |
| 201 | ZNF519 |  |  | 508 | 0.857 | 0.5756 | Yes |
| 202 | TTF2 |  |  | 517 | 0.853 | 0.5761 | Yes |
| 203 | BUB1B |  |  | 535 | 0.846 | 0.5748 | Yes |
| 204 | BIRC5 |  |  | 541 | 0.842 | 0.5759 | Yes |
| 205 | CEP152 |  |  | 547 | 0.834 | 0.5770 | Yes |
| 206 | CENPQ |  |  | 557 | 0.825 | 0.5772 | Yes |
| 207 | HIST1H2BH |  |  | 561 | 0.824 | 0.5787 | Yes |
| 208 | WDHD1 |  |  | 562 | 0.823 | 0.5808 | Yes |
| 209 | E2F2 |  |  | 576 | 0.814 | 0.5801 | Yes |
| 210 | GPC6 |  |  | 577 | 0.814 | 0.5822 | Yes |
| 211 | HMGB1 |  |  | 583 | 0.810 | 0.5832 | Yes |
| 212 | DIAPH3 |  |  | 585 | 0.808 | 0.5851 | Yes |
| 213 | MEIS1 |  |  | 588 | 0.807 | 0.5867 | Yes |
| 214 | FUS |  |  | 590 | 0.806 | 0.5886 | Yes |
| 215 | TK1 |  |  | 591 | 0.806 | 0.5906 | Yes |
| 216 | LCORL |  |  | 594 | 0.804 | 0.5923 | Yes |
| 217 | TRA2B |  |  | 596 | 0.802 | 0.5941 | Yes |
| 218 | STAG1 |  |  | 602 | 0.800 | 0.5951 | Yes |
| 219 | RAD18 |  |  | 603 | 0.799 | 0.5971 | Yes |
| 220 | CDK6 |  |  | 605 | 0.798 | 0.5989 | Yes |
| 221 | ARHGEF26 |  |  | 608 | 0.795 | 0.6005 | Yes |
| 222 | ACYP1 |  |  | 609 | 0.794 | 0.6026 | Yes |
| 223 | RBBP4 |  |  | 610 | 0.794 | 0.6046 | Yes |
| 224 | RFWD3 |  |  | 616 | 0.790 | 0.6056 | Yes |
| 225 | APBB2 |  |  | 617 | 0.790 | 0.6076 | Yes |
| 226 | E2F7 |  |  | 618 | 0.789 | 0.6096 | Yes |
| 227 | GEN1 |  |  | 619 | 0.789 | 0.6116 | Yes |
| 228 | SFMBT1 |  |  | 636 | 0.781 | 0.6103 | Yes |
| 229 | CENPL |  |  | 640 | 0.778 | 0.6116 | Yes |
| 230 | PBX3 |  |  | 657 | 0.771 | 0.6103 | Yes |
| 231 | PCDH17 |  |  | 658 | 0.770 | 0.6122 | Yes |
| 232 | HNRNPD |  |  | 659 | 0.769 | 0.6142 | Yes |
| 233 | NUP62 |  |  | 664 | 0.767 | 0.6153 | Yes |
| 234 | SKA3 |  |  | 668 | 0.765 | 0.6166 | Yes |
| 235 | C18orf54 |  |  | 669 | 0.765 | 0.6186 | Yes |
| 236 | KIF18A |  |  | 679 | 0.759 | 0.6186 | Yes |
| 237 | PFN2 |  |  | 680 | 0.759 | 0.6206 | Yes |
| 238 | FANCB |  |  | 681 | 0.759 | 0.6225 | Yes |
| 239 | DSEL |  |  | 683 | 0.756 | 0.6242 | Yes |
| 240 | CDC25C |  |  | 684 | 0.755 | 0.6261 | Yes |
| 241 | GAS2L3 |  |  | 685 | 0.755 | 0.6281 | Yes |
| 242 | CENPO |  |  | 686 | 0.755 | 0.6300 | Yes |
| 243 | KIF18B |  |  | 698 | 0.747 | 0.6296 | Yes |
| 244 | ARL6IP6 |  |  | 702 | 0.745 | 0.6309 | Yes |
| 245 | SMC3 |  |  | 708 | 0.743 | 0.6317 | Yes |
| 246 | PLK4 |  |  | 720 | 0.739 | 0.6313 | Yes |
| 247 | ATAD5 |  |  | 721 | 0.739 | 0.6332 | Yes |
| 248 | ANP32E |  |  | 722 | 0.739 | 0.6351 | Yes |
| 249 | MAD2L2 |  |  | 724 | 0.736 | 0.6368 | Yes |
| 250 | CTCF |  |  | 729 | 0.731 | 0.6378 | Yes |
| 251 | AGO2 |  |  | 739 | 0.727 | 0.6378 | Yes |
| 252 | FAT1 |  |  | 740 | 0.727 | 0.6396 | Yes |
| 253 | HAT1 |  |  | 741 | 0.727 | 0.6415 | Yes |
| 254 | CKAP5 |  |  | 752 | 0.723 | 0.6412 | Yes |
| 255 | PBRM1 |  |  | 759 | 0.719 | 0.6418 | Yes |
| 256 | CCDC150 |  |  | 773 | 0.713 | 0.6410 | Yes |
| 257 | CHEK1 |  |  | 779 | 0.709 | 0.6417 | Yes |
| 258 | CCDC15 |  |  | 782 | 0.709 | 0.6431 | Yes |
| 259 | CBX5 |  |  | 783 | 0.708 | 0.6449 | Yes |
| 260 | PSRC1 |  |  | 787 | 0.705 | 0.6461 | Yes |
| 261 | CENPC |  |  | 797 | 0.702 | 0.6460 | Yes |
| 262 | DTYMK |  |  | 800 | 0.700 | 0.6474 | Yes |
| 263 | POLH |  |  | 807 | 0.697 | 0.6479 | Yes |
| 264 | SOX6 |  |  | 813 | 0.696 | 0.6487 | Yes |
| 265 | RSRC1 |  |  | 818 | 0.693 | 0.6496 | Yes |
| 266 | ASAP1 |  |  | 823 | 0.689 | 0.6505 | Yes |
| 267 | BEST3 |  |  | 826 | 0.688 | 0.6519 | Yes |
| 268 | AASS |  |  | 830 | 0.687 | 0.6530 | Yes |
| 269 | TUBB |  |  | 836 | 0.685 | 0.6537 | Yes |
| 270 | ALYREF |  |  | 847 | 0.680 | 0.6534 | Yes |
| 271 | H2AFZ |  |  | 851 | 0.677 | 0.6545 | Yes |
| 272 | KLHL13 |  |  | 853 | 0.677 | 0.6560 | Yes |
| 273 | RDX |  |  | 858 | 0.675 | 0.6569 | Yes |
| 274 | RNASEH2A |  |  | 859 | 0.674 | 0.6586 | Yes |
| 275 | SKA1 |  |  | 868 | 0.672 | 0.6586 | Yes |
| 276 | ZNF92 |  |  | 871 | 0.670 | 0.6599 | Yes |
| 277 | DDB2 |  |  | 872 | 0.669 | 0.6616 | Yes |
| 278 | BRD7 |  |  | 883 | 0.667 | 0.6612 | Yes |
| 279 | SMARCA2 |  |  | 895 | 0.660 | 0.6607 | Yes |
| 280 | NEK2 |  |  | 901 | 0.659 | 0.6613 | Yes |
| 281 | MELK |  |  | 915 | 0.653 | 0.6603 | Yes |
| 282 | JADE1 |  |  | 931 | 0.647 | 0.6588 | Yes |
| 283 | H2AFV |  |  | 933 | 0.647 | 0.6603 | Yes |
| 284 | KIF20B |  |  | 934 | 0.647 | 0.6619 | Yes |
| 285 | PTTG1 |  |  | 936 | 0.647 | 0.6633 | Yes |
| 286 | ARX |  |  | 937 | 0.647 | 0.6650 | Yes |
| 287 | LIN54 |  |  | 938 | 0.646 | 0.6666 | Yes |
| 288 | HMGXB4 |  |  | 943 | 0.645 | 0.6674 | Yes |
| 289 | H3F3A |  |  | 944 | 0.643 | 0.6691 | Yes |
| 290 | GAD1 |  |  | 947 | 0.641 | 0.6703 | Yes |
| 291 | RTTN |  |  | 949 | 0.640 | 0.6717 | Yes |
| 292 | MDK |  |  | 950 | 0.640 | 0.6733 | Yes |
| 293 | TMEM237 |  |  | 952 | 0.639 | 0.6748 | Yes |
| 294 | CENPN |  |  | 953 | 0.638 | 0.6764 | Yes |
| 295 | DCLK2 |  |  | 957 | 0.638 | 0.6774 | Yes |
| 296 | CKS2 |  |  | 966 | 0.634 | 0.6773 | Yes |
| 297 | PRIM2 |  |  | 968 | 0.633 | 0.6787 | Yes |
| 298 | NPAT |  |  | 982 | 0.626 | 0.6776 | Yes |
| 299 | HAUS1 |  |  | 987 | 0.624 | 0.6784 | Yes |
| 300 | STAG2 |  |  | 993 | 0.622 | 0.6790 | Yes |
| 301 | FBXW7 |  |  | 1002 | 0.620 | 0.6789 | Yes |
| 302 | ZNF680 |  |  | 1004 | 0.619 | 0.6802 | Yes |
| 303 | CNTRL |  |  | 1011 | 0.617 | 0.6806 | Yes |
| 304 | TUBA1B |  |  | 1012 | 0.617 | 0.6821 | Yes |
| 305 | MIS18A |  |  | 1023 | 0.614 | 0.6816 | Yes |
| 306 | KCNJ2 |  |  | 1033 | 0.613 | 0.6813 | Yes |
| 307 | CDCA8 |  |  | 1043 | 0.610 | 0.6810 | Yes |
| 308 | CEP57 |  |  | 1053 | 0.607 | 0.6807 | Yes |
| 309 | BAZ1B |  |  | 1072 | 0.601 | 0.6785 | Yes |
| 310 | TTF1 |  |  | 1080 | 0.597 | 0.6786 | Yes |
| 311 | FIGN |  |  | 1093 | 0.594 | 0.6776 | Yes |
| 312 | ZNF90 |  |  | 1101 | 0.592 | 0.6777 | Yes |
| 313 | MSH2 |  |  | 1107 | 0.590 | 0.6782 | Yes |
| 314 | EFS |  |  | 1120 | 0.588 | 0.6772 | Yes |
| 315 | SMCHD1 |  |  | 1123 | 0.587 | 0.6783 | Yes |
| 316 | N4BP2 |  |  | 1124 | 0.586 | 0.6797 | Yes |
| 317 | KIF20A |  |  | 1133 | 0.583 | 0.6796 | Yes |
| 318 | SPATA5 |  |  | 1139 | 0.581 | 0.6800 | Yes |
| 319 | PDS5B |  |  | 1140 | 0.580 | 0.6815 | Yes |
| 320 | HMGB3 |  |  | 1154 | 0.577 | 0.6803 | Yes |
| 321 | NUCKS1 |  |  | 1178 | 0.570 | 0.6770 | Yes |
| 322 | DEPDC1 |  |  | 1179 | 0.569 | 0.6784 | Yes |
| 323 | ITGB3BP |  |  | 1180 | 0.568 | 0.6799 | Yes |
| 324 | ANP32B |  |  | 1187 | 0.566 | 0.6801 | Yes |
| 325 | HP1BP3 |  |  | 1198 | 0.562 | 0.6794 | Yes |
| 326 | POLE2 |  |  | 1199 | 0.561 | 0.6809 | Yes |
| 327 | RIF1 |  |  | 1204 | 0.560 | 0.6815 | Yes |
| 328 | PARPBP |  |  | 1210 | 0.560 | 0.6818 | Yes |
| 329 | E2F3 |  |  | 1223 | 0.555 | 0.6808 | Yes |
| 330 | EXOSC8 |  |  | 1230 | 0.553 | 0.6809 | Yes |
| 331 | OSBPL11 |  |  | 1232 | 0.552 | 0.6821 | Yes |
| 332 | HADH |  |  | 1242 | 0.548 | 0.6817 | Yes |
| 333 | CTDSPL2 |  |  | 1256 | 0.544 | 0.6804 | Yes |
| 334 | CEP135 |  |  | 1264 | 0.541 | 0.6803 | Yes |
| 335 | TOP2B |  |  | 1269 | 0.540 | 0.6808 | Yes |
| 336 | SRSF10 |  |  | 1289 | 0.535 | 0.6783 | Yes |
| 337 | PIP5K1B |  |  | 1290 | 0.535 | 0.6796 | Yes |
| 338 | BCL11B |  |  | 1291 | 0.534 | 0.6810 | Yes |
| 339 | HMGN5 |  |  | 1292 | 0.534 | 0.6824 | Yes |
| 340 | KIF2C |  |  | 1309 | 0.530 | 0.6804 | No |
| 341 | PIK3R3 |  |  | 1327 | 0.526 | 0.6782 | No |
| 342 | DIAPH1 |  |  | 1344 | 0.522 | 0.6763 | No |
| 343 | CENPI |  |  | 1351 | 0.519 | 0.6763 | No |
| 344 | FANCL |  |  | 1353 | 0.519 | 0.6774 | No |
| 345 | DLX6 |  |  | 1357 | 0.517 | 0.6781 | No |
| 346 | CCNB2 |  |  | 1373 | 0.513 | 0.6763 | No |
| 347 | LIN9 |  |  | 1376 | 0.512 | 0.6772 | No |
| 348 | NUP107 |  |  | 1388 | 0.509 | 0.6763 | No |
| 349 | HAUS6 |  |  | 1401 | 0.505 | 0.6751 | No |
| 350 | OPA1 |  |  | 1428 | 0.497 | 0.6710 | No |
| 351 | SP3 |  |  | 1438 | 0.495 | 0.6704 | No |
| 352 | LIN52 |  |  | 1442 | 0.495 | 0.6710 | No |
| 353 | FRYL |  |  | 1452 | 0.492 | 0.6704 | No |
| 354 | ARHGAP11B |  |  | 1471 | 0.488 | 0.6679 | No |
| 355 | EXOSC9 |  |  | 1472 | 0.487 | 0.6692 | No |
| 356 | DCLRE1C |  |  | 1497 | 0.480 | 0.6654 | No |
| 357 | CKS1B |  |  | 1502 | 0.479 | 0.6658 | No |
| 358 | HIST1H1A |  |  | 1533 | 0.473 | 0.6608 | No |
| 359 | KAT2B |  |  | 1537 | 0.472 | 0.6614 | No |
| 360 | IQGAP3 |  |  | 1546 | 0.471 | 0.6609 | No |
| 361 | HNRNPU |  |  | 1548 | 0.470 | 0.6619 | No |
| 362 | CCDC88A |  |  | 1564 | 0.466 | 0.6600 | No |
| 363 | DLEU2 |  |  | 1587 | 0.462 | 0.6567 | No |
| 364 | G2E3 |  |  | 1602 | 0.459 | 0.6549 | No |
| 365 | PDZRN3 |  |  | 1603 | 0.459 | 0.6561 | No |
| 366 | IFT122 |  |  | 1650 | 0.447 | 0.6477 | No |
| 367 | TUBB4B |  |  | 1659 | 0.444 | 0.6472 | No |
| 368 | NRXN3 |  |  | 1692 | 0.438 | 0.6417 | No |
| 369 | BRD8 |  |  | 1694 | 0.437 | 0.6426 | No |
| 370 | HNRNPL |  |  | 1747 | 0.428 | 0.6330 | No |
| 371 | GLCCI1 |  |  | 1764 | 0.425 | 0.6307 | No |
| 372 | PPP2R3C |  |  | 1800 | 0.418 | 0.6246 | No |
| 373 | ELF2 |  |  | 1806 | 0.416 | 0.6246 | No |
| 374 | ING3 |  |  | 1844 | 0.411 | 0.6180 | No |
| 375 | PCNP |  |  | 1846 | 0.411 | 0.6188 | No |
| 376 | SP1 |  |  | 1874 | 0.407 | 0.6143 | No |
| 377 | NCAPH2 |  |  | 1915 | 0.401 | 0.6071 | No |
| 378 | ARL4D |  |  | 1935 | 0.398 | 0.6042 | No |
| 379 | CENPW |  |  | 1959 | 0.395 | 0.6004 | No |
| 380 | ZEB2 |  |  | 2009 | 0.386 | 0.5913 | No |
| 381 | TMX1 |  |  | 2012 | 0.386 | 0.5918 | No |
| 382 | EPHA4 |  |  | 2034 | 0.382 | 0.5885 | No |
| 383 | MSI2 |  |  | 2042 | 0.382 | 0.5880 | No |
| 384 | TULP3 |  |  | 2059 | 0.379 | 0.5857 | No |
| 385 | XPO1 |  |  | 2091 | 0.374 | 0.5802 | No |
| 386 | ANLN |  |  | 2125 | 0.371 | 0.5743 | No |
| 387 | TSC22D2 |  |  | 2132 | 0.370 | 0.5740 | No |
| 388 | DLL1 |  |  | 2147 | 0.368 | 0.5721 | No |
| 389 | SESTD1 |  |  | 2157 | 0.367 | 0.5712 | No |
| 390 | NUP205 |  |  | 2169 | 0.366 | 0.5698 | No |
| 391 | ABAT |  |  | 2170 | 0.365 | 0.5708 | No |
| 392 | NGLY1 |  |  | 2174 | 0.365 | 0.5711 | No |
| 393 | HNRNPR |  |  | 2175 | 0.365 | 0.5720 | No |
| 394 | HNRNPDL |  |  | 2203 | 0.362 | 0.5673 | No |
| 395 | ST8SIA4 |  |  | 2207 | 0.361 | 0.5676 | No |
| 396 | LRBA |  |  | 2248 | 0.357 | 0.5603 | No |
| 397 | EFCAB2 |  |  | 2279 | 0.353 | 0.5550 | No |
| 398 | NUP155 |  |  | 2313 | 0.347 | 0.5491 | No |
| 399 | SKIDA1 |  |  | 2350 | 0.342 | 0.5425 | No |
| 400 | PAICS |  |  | 2403 | 0.334 | 0.5326 | No |
| 401 | SKA2 |  |  | 2445 | 0.329 | 0.5250 | No |
| 402 | CCNB1 |  |  | 2480 | 0.325 | 0.5188 | No |
| 403 | HNRNPA2B1 |  |  | 2523 | 0.320 | 0.5109 | No |
| 404 | TMEM245 |  |  | 2531 | 0.319 | 0.5103 | No |
| 405 | ENOSF1 |  |  | 2535 | 0.319 | 0.5105 | No |
| 406 | ATF7IP |  |  | 2544 | 0.317 | 0.5096 | No |
| 407 | SMAD4 |  |  | 2589 | 0.313 | 0.5013 | No |
| 408 | EAF1 |  |  | 2669 | 0.303 | 0.4858 | No |
| 409 | SRSF3 |  |  | 2732 | 0.297 | 0.4737 | No |
| 410 | HMGN1 |  |  | 2742 | 0.295 | 0.4726 | No |
| 411 | SMARCA5 |  |  | 2749 | 0.295 | 0.4721 | No |
| 412 | ARID1A |  |  | 2780 | 0.291 | 0.4667 | No |
| 413 | NUP160 |  |  | 2788 | 0.290 | 0.4660 | No |
| 414 | BUB3 |  |  | 2806 | 0.288 | 0.4632 | No |
| 415 | ATRNL1 |  |  | 2823 | 0.287 | 0.4606 | No |
| 416 | SAE1 |  |  | 2872 | 0.283 | 0.4514 | No |
| 417 | CCND2 |  |  | 2876 | 0.282 | 0.4515 | No |
| 418 | ODF2 |  |  | 2911 | 0.278 | 0.4452 | No |
| 419 | CNTROB |  |  | 2918 | 0.277 | 0.4447 | No |
| 420 | ZC3H12C |  |  | 2952 | 0.274 | 0.4386 | No |
| 421 | OTUD3 |  |  | 2976 | 0.272 | 0.4345 | No |
| 422 | SIN3A |  |  | 2981 | 0.271 | 0.4344 | No |
| 423 | COX8A |  |  | 2998 | 0.270 | 0.4318 | No |
| 424 | PTPRE |  |  | 3014 | 0.267 | 0.4293 | No |
| 425 | SP2 |  |  | 3019 | 0.267 | 0.4292 | No |
| 426 | HNRNPH1 |  |  | 3026 | 0.266 | 0.4286 | No |
| 427 | KIAA1328 |  |  | 3035 | 0.265 | 0.4276 | No |
| 428 | STARD9 |  |  | 3044 | 0.264 | 0.4267 | No |
| 429 | NRF1 |  |  | 3093 | 0.261 | 0.4174 | No |
| 430 | GRIK3 |  |  | 3138 | 0.256 | 0.4090 | No |
| 431 | RALY |  |  | 3167 | 0.254 | 0.4038 | No |
| 432 | PID1 |  |  | 3215 | -0.253 | 0.3948 | No |
| 433 | MYO1B |  |  | 3217 | -0.254 | 0.3952 | No |
| 434 | KITLG |  |  | 3280 | -0.262 | 0.3831 | No |
| 435 | RAP2B |  |  | 3360 | -0.276 | 0.3675 | No |
| 436 | TMEM126B |  |  | 3502 | -0.300 | 0.3391 | No |
| 437 | TPM4 |  |  | 3591 | -0.314 | 0.3218 | No |
| 438 | STAM2 |  |  | 3618 | -0.318 | 0.3172 | No |
| 439 | SEPT10 |  |  | 3805 | -0.350 | 0.2797 | No |
| 440 | TUBB6 |  |  | 3904 | -0.368 | 0.2604 | No |
| 441 | CLIC4 |  |  | 4075 | -0.404 | 0.2263 | No |
| 442 | COPS8 |  |  | 4137 | -0.419 | 0.2148 | No |
| 443 | RPS10 |  |  | 4340 | -0.485 | 0.1743 | No |
| 444 | BAHCC1 |  |  | 4458 | -0.523 | 0.1515 | No |
| 445 | PIP4K2A |  |  | 4591 | -0.581 | 0.1257 | No |
| 446 | EIF3D |  |  | 4676 | -0.631 | 0.1099 | No |
| 447 | HERPUD1 |  |  | 4762 | -0.687 | 0.0941 | No |
| 448 | SLC38A1 |  |  | 4773 | -0.694 | 0.0938 | No |
| 449 | STK17B |  |  | 4824 | -0.734 | 0.0854 | No |
| 450 | CLIC1 |  |  | 4899 | -0.809 | 0.0722 | No |
| 451 | IGF2BP1 |  |  | 5048 | -1.029 | 0.0442 | No |
| 452 | RND3 |  |  | 5128 | -1.230 | 0.0310 | No |
| 453 | ARL4C |  |  | 5143 | -1.279 | 0.0314 | No |
Table: GSEA details [plain text format]

  

Fig 2: RICHARDS\_NOWAKOWSKI\_SCIENCE\_2017\_MGE\_IPC2\_UPREG: Random ES distribution      
 Gene set null distribution of ES for **RICHARDS\_NOWAKOWSKI\_SCIENCE\_2017\_MGE\_IPC2\_UPREG**
